# Putting them on a strong spiritual path: Indigenous doulas responding to the needs of Indigenous mothers and communities

**DOI:** 10.1186/s12939-021-01521-3

**Published:** 2021-08-26

**Authors:** Jaime Cidro, Caroline Doenmez, Stephanie Sinclair, Alexandra Nychuk, Larissa Wodtke, Ashley Hayward

**Affiliations:** 1 University of Winnipeg, 515 Portage Avenue, Winnipeg, Manitoba R3B 2E9 Canada; 2 University of Manitoba, 66 Chancellors Cir, Winnipeg, Manitoba R3T 2N2 Canada

**Keywords:** Indigenous doulas, Culturally informed care, Medical racism, Harm reduction, Advocacy, Resurgence, Birthing sovereignty

## Abstract

**Objective:**

In the past few years, increasing numbers of Indigenous doula collectives have been forming across Canada. Indigenous doulas provide continuous, culturally appropriate support to Indigenous women during pregnancy, birth, and the post-partum period. This support is critical to counter systemic medical racism and socioeconomic barriers that Indigenous families disproportionately face. This paper analyzes interviews with members of five Indigenous doula collectives to demonstrate their shared challenges, strategies, and missions.

**Methods:**

Qualitative interviews were conducted with members of five Indigenous doula collectives across Canada in 2020. Interviews were transcribed and returned to participants for their approval. Approved transcripts were then coded by all members of the research team to ascertain the dominant themes emerging across the interviews.

**Results:**

Two prominent themes emerged in the interviews. The first theme is “Indigenous doulas responding to community needs.” Participants indicated that responding to community needs involves harm reduction and trauma-informed care, supporting cultural aspects of birthing and family, and helping clients navigate socioeconomic barriers. The second theme is “Indigenous doulas building connections with mothers.” Participants’ comments on providing care to mothers emphasize the importance of advocacy in healthcare systems, boosting their clients’ confidence and skills, and being the “right” doula for their clients. These two inter-related themes stem from Indigenous doulas’ efforts to counter dynamics in healthcare and social services that can be harmful to Indigenous families, while also integrating cultural teachings and practices.

**Conclusion:**

This paper illustrates that Indigenous doula care responds to a wide range of issues that affect Indigenous women’s experiences of pregnancy, birth, and the post-partum period. Through building strong, trusting, and non-judgemental connections with mothers and responding to community needs, Indigenous doulas play a critical role in countering medical racism in hospital settings and advancing the resurgence of Indigenous birthing sovereignty.

## Introduction

Indigenous doulas provide culturally appropriate support to Indigenous women during pregnancy, birth, and the post-partum period. Doulas extend their role of emotional support companion to advocacy that connects women to various social supports following the birth. Doulas maintain boundaries within the medical birthing experience and empower Indigenous women to create a positive experience for themselves. These doulas also experience personal transformation that “nourish[] [them] through this training and practice” ( [1] p.5). This paper is a part of a larger project developing and piloting an urban Indigenous doula program in Winnipeg, Manitoba, Canada. The research question that is guiding this component of the project is: how do other Indigenous doula collectives deliver their services in terms of administrative, technical, cultural, and emotional support for Indigenous birth workers? We conducted five interviews with five Indigenous doula collectives in Canada, including one in Winnipeg, to identify the ways in which they provide service and support to Indigenous women and families. This paper describes two of the dominant themes that emerged out of these interviews: (a) responding to community needs and (b) connections with mothers.

## Background

The Indigenous doula is not a new role. Its linguistic roots are in Greek, however, there is a distinction between the root word “douleía,” which translates to “slavery,” and “doula,” which has come to mean “mother’s assistant” [2]. Some scholars credit American anthropologist Dana Raphael as the first to adopt the term doula in the context of a support companion for labouring women [3, 4]. The contemporary doula refers to the growing occupation of women supporting other women during pregnancy, labour, birth, and post-partum. Historically, family members or experienced local women provided support as birth workers [5]. The more formalized doula role, as we know it today, is the result of women living away from their families, and in the case of First Nations women, being forced to birth away from their home communities and family. A doula provides continuous physical, emotional, and advocacy support during labour and birth, but does not provide medical, midwifery, or nursing care [5]. Mainstream doula care is available widely, especially in urban areas, but it is also expensive, making it inaccessible for many low-income families. Mainstream doulas tend to be “predominantly white, well-educated, married women with children, living in urban areas, in upper-middle-income households,” which speaks to a paucity of diversity in doula service provision ([6] , p. 774). Indigenous doulas differ from these mainstream doula care providers because the care is grounded in culture and spirituality and recognizes the sacredness of women as life-givers and water carriers. Indigenous doulas have become increasingly prevalent across Canadian Indigenous communities, both urban and rural/remote. They are partially a resurgence of Indigenous cultural knowledge, but also a reflection of the resistance of long held medical practices that have served to disenfranchise traditional approaches to pregnancy and childbirth.

Indigenous women have traditionally delivered their babies at home surrounded by local midwives and family members [7]. The knowledge about birthing infants was transmitted inter-generationally and included not only physical logistics of birthing, but also traditional medicines to deal with a range of issues associated with delivery and post-partum [7]. The move from home to hospital births has had a profoundly negative impact on Indigenous women’s birthing experiences. The impact of the birth experience, including support or lack thereof, lasts for many years as women recount the story of their labour and the birth of their children long after the birth [8]. Findings from the Cochrane review [9] show that positive birth outcomes are more prevalent when the support provider is not a member of the hospital staff, demonstrating the importance of trained doulas.

A 2010 report by Bowser and Hill reveals that many women around the globe feel disrespected and abused during facility-based childbirth, experiences which include “subtle humiliation of women, discrimination against certain sub-groups of women, overt humiliation, abandonment of care and physical and verbal abuse” ([10] p. 3). The normalization of facility- and hospital-based births has led to the overmedicalization of birth and the authority of Western-based medical professionals overtook that of long-practiced birth knowledge in marginalized communities. Black communities, like many Indigenous communities, experienced the criminalization of traditional birth work, which resulted in the diminishment and disappearance of these culturally relevant practices [11, 12]. Often doulas are the only source of culturally competent care for expectant Indigenous mothers during childbirth ([6] p. 777–778). Studies of midwives in Mexico [13] and the US [14, 15] provide examples of successful, culturally appropriate maternal care programs that have been driven by community to empower participants and improve the wellness outcomes of both mothers and babies [13–15]. According to research with First Nations communities in British Columbia, the positive aspects of relationships with care providers are underpinned by “respect, understanding of cultural context and connection with communities” ([16] p. 4).

Indigenous women in Canada face less desirable birth outcomes compared to other groups [17]. For example, Statistics Canada [18] reports that Indigenous infants have overall higher rates of adverse birth outcomes than the rest of Canadian infants. Specifically, they note that Inuit infants have the highest rates of preterm birth (11.4%) compared to non-Indigenous infants in Canada at 6.7% [18]. Statistics also show that Indigenous infants tend to be large for gestational age at birth when (18.8%) compared to non-Indigenous infants (10.6%) [18]. Furthermore, the rate of postneonatal death for Indigenous babies is reported as 4.8 for every 1000 surviving births compared to 1.1 for every 1000 surviving births for non-Indigenous babies [18]. Disparities in maternal wellness in Canada are intertwined with colonization, which has created deeply rooted inequalities in socioeconomic status and health outcomes between Indigenous and non-Indigenous people. Socioeconomic factors that impact Indigenous women’s pregnancy experiences and birth outcomes include: reduced access to standard prenatal care; inaccurate estimation of gestational age and subsequent complications of post-term pregnancies; pre-existing medical conditions; young maternal age; marital status; malnutrition; and low educational attainment [19–22]. These factors speak to the ongoing social and economic structural inequalities that disproportionately harm Indigenous people.

A review of the Canadian Indigenous Women’s Perspectives of Maternal Health and Health Care Services conducted in 2016 argues that “the existence of health inequities between Indigenous and non-Indigenous women in urban areas reinforces the need to address structural barriers to health” ([23] p. 343). As historians McCallum and Perry [24] contend, healthcare systems that do not address racism “implicitly re-centre and privilege whiteness as the normative perspective while failing to address the myriad ways that racism deprives people of opportunities and structures their lives” (p. 13). In 2020 Canadians were able to see how insidious racism is within the healthcare system through the death of Indigenous woman Joyce Echaquan in a hospital in Quebec while she was told by hospital employees that she was “stupid, only good for sex, and that she would be better off dead” [25]. As an intervention for this systemic racism, Indigenous doulas provide culturally appropriate advocacy to Indigenous women during pregnancy, birth, and the post-partum period [1].

The Truth and Reconciliation Commission’s Calls to Action (18–24 specifically) acknowledge that “the current state of Aboriginal health in Canada is the direct result of previous Canadian government policies,” and therefore, call for all levels of government to acknowledge structural conditions that contribute to overall wellness of Indigenous peoples ([26] p.2). Our urban Indigenous doula program responds to Call 19, which demands that Canadians “establish measurable goals to identify and close the gaps in health outcomes between Aboriginal and non-Aboriginal communities.... Such efforts would focus on indicators such as: infant mortality, maternal health” ([26] p.2). Furthermore, our project is a substantial step towards Indigenous birthing sovereignty, defined here as Indigenous women’s abilities to self-determine how and where they give birth and receive culturally appropriate care throughout pregnancy, birth, and the post-partum period.

## Methodology

In 2020 we interviewed five Indigenous doula collectives across Canada using the “conversational method” [27], which is often considered an Indigenous research method and through which we discussed their experiences in establishing Indigenous doula collectives, their service delivery models, and their approaches to training and fee payment structures. We identified these groups using various Internet and social media searches. Doula collective models vary, but in essence, they are a group of doulas who provide support to pregnant women. They may have one doula support one expectant mother or have multiple doulas supporting the same family. In some cases, the doulas are on a salary, but in most cases the doulas are paid per birth. One Manitoba group that was interviewed is a partner on an existing project called “Indigenous Doulas for First Nations Women Who Travel for Birth.”

The research underwent research ethics review from the University of Winnipeg Human Research Ethics Board. All participants consented orally to the project and were provided with an honorarium and gift for participating. The first interview took place in person, and the remaining interviews were held over Zoom due to COVID restrictions on travel. The interviews were audio-recorded and varied from one hour to four hours. One interview was done with one representative from each of the five doula collectives. Interviews were always recorded by at least two of the interviewers to ensure that, if one recording was flawed or damaged, an alternative copy was available. Interviews were transcribed verbatim and sent to the participants for approval. They were given several weeks to send feedback to the research team with any edits they wanted made to their transcripts. Once transcripts had been approved by the doulas, the researchers developed a coding framework as a group using the constant comparative method and drawing from grounded theory [28–30] to identify recurring themes. This method ensures that “the nuances in meaning brought by multiple researchers adds richness to the analysis by prompting deeper analysis” ([30] p. 26). The research team is comprised of almost all Indigenous scholars and graduate students. Some of the research team attended all of the interviews and all of the research team participated in coding and analysis of the transcripts. We come from a range of academic backgrounds (for example, Anthropology, Native Studies, Peace and Conflict Studies) and used these disciplinary lenses, as well as our own experiences as parents and women, as we developed individual coding frameworks. The lead author (JC) compiled the frameworks and developed a common coding framework that was agreed upon by the research team. Independently, the research team coded the transcripts using a selective approach to ascertain codes that were frequent and most pertinent to the original research question [31]. The research team then came together to compare their coded transcripts and adjusted a master transcript. All inconsistencies were discussed, which required a “sensitivity to differences between emerging concepts/categories” ([29] p. 515). Based on the analysis of the transcripts, the research team was able to determine which codes were most dominant. A draft of this paper was also sent to the participants to ensure that the experiences of the doula collectives were accurately reflected. Member checking [31] is important to ensure that our interpretation of their experiences is valid.

## Results

Two dominant themes emerged from the transcripts. The first theme is “responding to community needs.” It refers to high community needs such as socioeconomic issues of housing, poverty, transportation, and mental health. The second theme is “connections with mothers,” which refers to participants describing how they build trust with their clients, how they reduce fear of systems and the birthing experience, and how they support mothers and families dealing with child and family services. These two themes are inter-related and in essence describe the ways that the Indigenous doulas work to circumvent systems that are negative and harmful to Indigenous women and families in healthcare and social services. This rationale is unsurprising because we see that the emergence of Indigenous doulas is partly in response to the systemic racism experienced in health and social services along with the high number of Indigenous children in care. As of March 31, 2018, there were 10,300 children in Manitoba Child and Family Services, and 90% of those children are noted as Indigenous [32].

## Responding to community needs

At the core of the work of Indigenous doulas are the communities that they serve and support. Continually adapting and modifying their service delivery model in ways that directly respond to the needs of the community is essential. Three subthemes that emerged from the interviews include: (i) harm reduction and trauma-informed care, (ii) supporting cultural aspects of birthing and family, and (iii) socioeconomic barriers.

### Harm reduction and trauma-informed care

Indigenous women who use drugs or alcohol are often reluctant to disclose their pregnancy to a healthcare provider based on fears of apprehension from child and family services. The doula collectives discuss the importance of being not only “non-judgemental” but also of providing a safe space where their clients are aware of their options if they choose to stop or minimize using alcohol and/or drugs or engaging in behaviour that would be deemed “high risk.” While not all doulas describe mothers with addictions as being highly prevalent in their work, some describe addiction as one component that requires important consideration. One participant describes how she ascertains whether a potential doula would embrace a harm reduction approach to care:

Harm reduction is hard for a lot of people. It’s really hard to support women who are drinking or who are using through their pregnancy without being like "oh well you need to quit doing meth and you need to quit drinking."We would know that, but we would never tell somebody "you need to quit drinking and you need to stop doing drugs." With every community training that we've done, we've revised our curriculum every single time based on harm reduction.

We know that across Canadian communities addictions have taken on a new level of complexity with narcotics such as methamphetamines and opioids [33], and our participants identify this need for doulas who are rigorously trained in a harm reduction approach. In some cases, it is dangerous for the doulas themselves to engage with the expectant mothers. One participant describes these challenges for doulas:

In the past two years it’s [methamphetamine] kind of exploded a lot more. When we're dealing with meth, most of them [mothers] really don't want to work on their addictions. We will try to support them but a lot of times it’s just like a breakdown, like it’s not going to happen. I think we've had seven instances where it’s like this isn't going to happen. We're going to meet with them, and they are really high. Then it becomes a danger to our staff as well because it’s really unpredictable. If they're smoking weed and they're high on weed, it’s not a big deal. We've had some moms and we've gone to work with them and they're drunk. It could be a safety issue, much less likely than when they're on meth.

Harm reduction and trauma-informed care are often challenging not only to conceptualize, but also to employ as a non-judgemental support person. For doulas who focus on incorporating traditional cultural activities into their work, it can be challenging to incorporate sacred medicines and teachings while a person is still an active user.

The role of Indigenous doulas in harm reduction and trauma-informed care is an important distinction between doulas and other birth workers, such as midwives. One participant notes the tension arising from doulas being seen as replacing the services provided by midwives. The recent increased focus on doulas may be perceived as problematic because it means that there is potentially less focus on training Indigenous people to be midwives; however, as this participant observes, the role of the doula is much different from a midwife, and she considers doulas as a valuable support in the interim while more Indigenous people are trained as midwives. In this sense, the participant uses the term “harm reduction” to refer to the role Indigenous doulas play in lessening harms to Indigenous people inflicted by healthcare systems:

I really just see the work we do as harm reduction. Because it's not realistic that we will be able to train 200 Indigenous midwives in two years, but we could train 200 Indigenous doulas. We could have an Indigenous care provider at every birth, much sooner than we could have an Indigenous midwife at every birth. In that sense I just really see it as harm reduction.

While, for the previous participant, “harm reduction” signifies supporting Indigenous people to reduce the impacts associated with their addictions, for this participant, the concept of “harm reduction” instead speaks to the importance of reducing the harms caused by healthcare systems themselves. The latter participant’s comment directs our attention to the fact that healthcare spaces are experienced as unsafe by many Indigenous people. Utilizing both understandings of harm reduction and a trauma-informed approach is essential for doulas who work with clients who may be deemed “high risk.” As opposed to stigmatizing or punishing people for their addictions, participants note that Indigenous doulas have critical roles to play in providing non-judgmental care and improving their clients’ experiences in hospitals. The doulas also describe how socioeconomic factors, trauma, and self-harm are cyclical in nature, and how they require a mindset of openness and non-judgement.

One participant describes how their doula collective members often feel ill-prepared to deal with complex mental health issues facing their clients. To address this issue, this collective ensures that doulas are well-connected to local resources so that they can refer mothers and follow up appropriately. She observes that it is often the women who need the help the most due to mental health and addictions who may not actively seek the care of a doula:

Somebody who's already got issues, those aren't the people who come to us. Obviously, those are the women that we do want to help the most, but we just find ourselves unprepared to help. What do we do if she's making some threats against herself? Every mom in that due month, is in a crazy state, "I'm ready to have my baby," when you have somebody with a mental health issue who is at that crazy point, it gets a little bit scary very quickly. We found ourselves unprepared. I'm on the phone with local services. We want to figure out the community health program’s protocol. How do we fall in line with that, to send them there or to make recommendations for programs? Because I don't think we can be the experts on that. We have many women like that and we're just not seeing them.

It is notable that these doulas feel that they may not even be reaching the populations most in need of harm reduction and trauma-informed care.

### Socioeconomic barriers

Participants explain the importance of doulas as being more than support for pregnancy and birth, essentially as “life doulas.” The need for “life doulas,” or someone who offers wholistic support, often stems from complex, inter-related socioeconomic issues such as addictions, poverty, and lack of adequate housing. For the doula collective operating in an urban centre, one participant describes some of the housing issues facing clients:

What was really unexpected, because the majority of our clients are urban, was the amount of multiple families in one house. We expected that maybe they live with their mom or dad, or maybe they live with a sibling. But it is just huge extended families. It really becomes situations that are out of control for the moms that we work with. They can't control who's drinking in their home or who's doing whatever. Even though they are personally doing good, that whole environment is not good.

Poverty impacts the clients and how the doula is able to support these clients. One participant describes how “food shortages and transportation” are so pertinent to the experiences of the mothers and subsequently impact the care that the doula can provide. As one participant recounts: “it’s not necessarily an issue *between* the doula and the client, but it does make for a lot of dropped appointments, which we still pay the doulas for, and it makes for a lot of difficult situations where we need to say no. When we cannot drive someone, or we cannot buy them additional food if they’ve gone through the program already.” There is a delicate balance between providing support for these complex socioeconomic issues and supporting women to develop coping mechanisms and skills. One participant explains how the doulas in her group struggle with defining and maintaining boundaries:

One thing we really have to continuously work on with our moms is boundaries because they will push boundaries all the time. They're phoning “can you pick me up and take me to my boyfriend's house?” Or in some cases, it’s three o’clock in the morning and there is a domestic violence situation and we say, “you know you should be phoning the police.” One of the ways that we're going to address that we're developing an information sheet for the moms that say “this is what we can do for you,” as opposed to a free-for-all kind of thing.

In many cases, the addictions and harmful behaviour of mothers and/or their loved ones impact their socioeconomic situations and vice versa as a result of the trauma and inequities inflicted by colonization. Therefore, it is necessary for doulas to understand this interplay of factors and respond holistically while maintaining their own self-care.

### Supporting cultural aspects of birthing and family

Our participants highlight the obligation to work in systems that are primarily run by non-Indigenous people who typically have limited awareness of the importance of culture and the sacredness of the bond between mother and child. In clinics, hospitals, and birth centres there may be some cursory awareness of Indigenous traditions from the region in which they are situated; however, the participants note that a deep understanding of the ways in which culture and tradition can be practiced as part of the birth experience is limited. One of the participants illustrates the instructions her collective received from grandmothers:

Most people who work in healthcare are non-Indigenous. It’s really good for them to have that support of another Indigenous person, and you know it’s important to include those cultural and traditional aspects of it. That’s really what we heard from the grandmothers. If we can put the cultural pieces and traditional knowledge back in birth, then that’s where you would restore that bond during pregnancy, and you would really clarify a woman's responsibility to her unborn child. If we could increase that bond or strengthen that bond during pregnancy, these other health issues that come after birth you know would disappear essentially. If we wanted our children to be healthier, then that has to start when they're in uterus. By bringing those cultural pieces and rights of passage back into birthing, we're ensuring that every child that’s born with that support begins their journey guided by spirit. They're already grounded, and they already have a spiritual connection right from birth, instead of being very clinical and very western. It puts them on a path where those connections might not be as strong.

Indigenous birth workers may or may not have an extensive cultural background, depending on their own life journeys. For those in an urban community, it is more difficult to have nation-specific traditional teachings, so it is important for the doulas to be flexible and to work with a common purpose of supporting women in their own cultural resurgence. As one participant states: “we wanted something radically different; we are about Indigenous reproductive justice, we are about decolonization, we are about resurgence.” Our participants talk about how they approach traditional culture with their clients through their own positionality. One participant who lives outside of her traditional territory explains:

My teachings haven’t just come from Cree and Anishinaabe systems, but also a lot of urban elders and people who are from different nations. I think that one of the ways that we negotiate that is just through being really upfront about who we are, where we come from, where our teachings come from, naming our teachers, and when we share something where that came from, and just being really upfront about it. We’re just sharing this with you, you can take it or leave it kind of thing.

Transparently integrating cultural approaches to doula care is important for working with mothers to reclaim or strengthen the bonds they have with their babies. Culture is a reminder about their responsibilities as parents and community members and their positive Indigenous identity.

## Connections with mothers

This second major theme relates to the ways in which doulas connect with the mothers they support. Factors such as systemic racism, fear of medical and social services, and a long history of interactions with child and family services require doulas to build trust with their clients. In some cases, shared experiences of being an Indigenous mother help to build this trust. The three subthemes that are most prevalent include: (i) advocacy in healthcare systems, (ii) boosting confidence and skills, and (iii) being the “right” doula.

### Advocacy in healthcare systems

Doulas are familiar with the type of medical appointments and exams that their clients are required to attend. One of the important parts of their role is navigating healthcare systems that are often hostile to Indigenous people and advocating for the mothers. One participant recounts the healthcare experiences of the mothers with whom the doulas work:

A lot of the times they are very shy and very passive and so you know when things are happening in hospital or even in medical appointments. They [mothers] don't speak up for themselves and they don't advocate for themselves. There’s lots of assumptions that are made: that they're alcoholics or drug addicts.

Another participant describes how the doulas need to be assertive on behalf of their clients when working in the hospital setting during a delivery. This example is particular to Quebec where physicians are more likely to speak French. She illustrates one example of this advocacy:

Some doctors are less acknowledging [of the role of doulas]. I've asked that at a birth they speak English to let the mom know what they're doing. In this case they were very rude because they're only speaking to each other in French but she can't understand. I said, “Can you let her know what's going on?” They got annoyed and spoke to each other only in French. Then I had to say, “My client is a little bit concerned can you let her know what's going on?”

The participant goes on to explain the ways in which the doulas act as advocates to circumvent the discrimination that the mothers experience not only in the clinic and hospital environment, but also through the complex systems of child welfare. This support is also about encouraging mothers to be assertive and actively engage in their own medical appointments. One participant details this empowerment:

We're able to be that buffer and say, “Wait a minute, this isn't acceptable to be speaking to her this way or treating her this way.” We really try to empower the women to speak up for themselves, we'll work with them. We will say, “Okay, you had a prenatal appointment; do you have concerns? Like, what do you want to talk about while you're there?”

This encouragement and modelling of health advocacy are some of the ways that doulas support and train Indigenous women to advocate for their own health within a Western system. These comments are also consistent with those made by American doulas of colour who express their health advocacy and empowerment for expectant mothers of colour as “holding space” ([6] p. 777).

### Boosting confidence and skills

Participants describe the importance of building skills like assertiveness, but also more general confidence and self-esteem. These attributes are important assets for the clients because they are powerful in situations and life stages beyond pregnancy and birth. For many of the Indigenous mothers, being a part of systems such as child and family services and experiencing trauma have impacted their ability to trust even themselves. As one participant asks: “how do you navigate through their many layers of trauma? You know it’s trust issues, it’s addictions, it’s sexual and physical abuse, it’s neglect, so there’s those things.” Due to lack of access to the adequate resources, Indigenous women often do not recognize the importance of feeling able to make decisions about your own body. One participant reveals how the doulas in her collective work to build the confidence and skills of their clients in relation to trauma:

Some of them [mothers] get there quickly, and then others are a lot of work. There's just so much toxicity and trauma that they don't realize that they can make choices. Or they don't have the resources or the knowledge. We tried to equip them with those resources and equip them with that knowledge.

The doulas themselves become those essential resources and sources of knowledge for the expectant mothers whom they support. They work to balance between simply delivering the information and providing mothers with the skills to be able to find those resources on their own.

### Being the “right doula”

The doula collectives have various mechanisms to connect prospective clients to doulas. In some cases, the doula collectives use paperwork and forms to “match” doulas with expectant mothers, and in other cases, they utilize community events where doulas and mothers can meet each other. One participant describes the format for the doulas in the collective to meet with potential clients:

It was like speed dating. We brought them [mothers and families] together with all of our doulas once we were ready to provide support and they were able to ask questions. The doulas rotated around the room and visited with each of the families so that they have an idea of who they might connect with. Then the families chose their doulas that way.

Similarly, another participant illustrates the significance of these types of meetings and being “known” in the community as a doula:

We had five doulas. The doulas went around and met with mothers for a set amount of time. That's for us what a consultation looks like. It’s like “hey how are you and do we mesh, do we meet, are we on the same page?” It was just like this great experience of crazy speed dating. It's out in the community, there's lots of community events, and between the five of us [doulas], we're always at stuff, and a lot of people know us now and just come ask.

Despite efforts to match doulas and expectant mothers appropriately, the doulas recognize that they are not always the right “fit” for the expectant mother and find ways to ensure that the match is good for everyone. One participant discusses her experiences of meeting with a teenage expectant mother and the realization that she did not connect with her:

I think all of us came to the work because we wanted to, we have this really strong value around every Indigenous family who wants a doula should have one. Then as you start to do the work you realize that I’m not everyone’s doula. That’s just like a piece of humility. I realized that I am not, because I’ve worked in programs before that are for youth, and there are people who are amazing at connecting and working with youth. I am not that person. I’m just awkward and they clearly think I’m awkward. After that meeting I didn’t end up working with that person, not because I didn’t think she was deserving of care, but just because I could tell that she wasn’t comfortable with me. It was just not going to be what she needed. She also wasn’t responding to anything, so it was just really obvious. I just made the call.

The doulas build trust with their clients as health advocates and sources of knowledge and skill development, staying cognizant of whether they are a good “fit” with their clients. Without this critical connection between mother and doula, supporting pregnancy, birthing, and post-partum effectively can be difficult. The following discussion provides some further analysis of the experiences of these doula collectives.

## Discussion

Indigenous doulas are a part of an important resurgence and reclamation of the sacred time during pregnancy, birthing, and post-partum. As the number of Indigenous children in care continues to escalate across the country and the cases of systemic racism within medical, justice, and social service systems become increasingly visible, the need for Indigenous doulas becomes more acute. The overrepresentation of Indigenous children in child welfare systems is a country-wide issue. In Manitoba, for example, Indigenous children make up almost 90% of children in CFS care [34]. The role of doulas in supporting pregnant Indigenous women who themselves may have had direct experience being in CFS custody or having other children in care has been taken up by the provincial government of Manitoba through a Social Impact Bond program [35]. This project, called “Restoring the Sacred Bond,” matches doulas with expectant Indigenous women who are considered “at risk” of having their infant apprehended through CFS.

The participants in our interviews describe the experiences of their doula collectives in supporting Indigenous women and families during pregnancy, birthing, and post-partum as essentially “softening” the negative experiences they face in Western systems and providing them with the space to embrace and incorporate traditional approaches into their pregnancy and birth. These well-documented negative experiences are rooted in colonization and the resultant socioeconomic and health disparities facing Indigenous communities [10]. The interview participants talk about the over-crowded housing, the high rates of addictions, and the vicarious violence that directly impact the ability of the expectant mothers to advocate for themselves and exercise control over their lives. These structural barriers to health described by Kolahdooz et al. [23] and the ways in which healthcare systems perpetuate racism [24, 25] are evident in the experiences of the doulas who support Indigenous women in Western healthcare systems. The experiences of the mothers receiving care from doulas is consistent with the 2010 Bowser and Hill report describing both subtle and overt humiliation and abuse of women and an abandonment of care [10].

The connection and relationship between doula and mother are essential to providing effective support not only to advocate against racism and build skills and confidence in clients, but also to ensure that the care provided uses a trauma-informed and harm reduction approach. One participant describes how they feel ill-equipped to provide support to women who struggle with vast mental health and addictions issues, and as a result, became well-informed of the community resources available. Another doula collective explains how many of their participants deal with complex socioeconomic issues and their role is to support mothers in a non-judgemental way to advocate for themselves and make decisions and plans for their pregnancy and birth.

What is distinctive about the support provided by the Indigenous doulas compared to mainstream doulas is the inclusion of traditional culture. The culturally appropriate maternity care that these Indigenous doulas provide is consistent with research done in Mexico by Tucker et al. [13] as well as in the US by Richards and Mousseau [14] and Prater and Davis [15]. Similarly, Varcoe’s [16] work on the relationships of care providers with Indigenous women is based on the role of cultural context and the connection with communities. This form of cultural competency and care underpins the work of the doulas.

The Indigenous doula collectives were formed in response to needs expressed by Indigenous women and communities. Having women emerge from their experiences of pregnancy and birth as feeling more connected to their sacred responsibilities as a parent is the larger goal of Indigenous doulas [1]. They express the values of birthing sovereignty and cultural resurgence as supporting their important work.

The main limitation for this project is the small number of Indigenous doula collectives that we interviewed. We conducted one interview with only one representative of each of the five Indigenous doula collectives, which are primarily located in urban or close to urban centres. None of the interviewed doula collectives are situated in remote communities. We expect that there are more Indigenous doula collectives emerging across North America, and as we continue with our urban Indigenous doula project, we will undertake an environmental scan to ascertain the different groups that exist. At this stage, we wanted to interview only those in the Canadian context, recognizing that there could be great opportunities to learn from other Indigenous doula collectives worldwide.

## Conclusion

Indigenous women are both at their most powerful and most vulnerable when they are pregnant. At their most powerful they are confident and equipped with knowledge both from their own Indigenous traditions and from the Western medical systems with which they need to engage. They are also vulnerable because of the complex socioeconomic factors that shape their daily experiences.

In interviews with five Indigenous doula collectives in Canada, several themes emerged, and the focus of this paper is on two of these themes: responding to community needs and connections with mothers. It is clear through these interviews that Indigenous doulas come to this work because of their awareness and engagement with the community as well as their deep commitment to working and connecting with Indigenous mothers and families. While undertaking this work, our participants discuss the factors that affect the way they need to respond to their communities and connect with the mothers. In responding to community needs, they note the importance of taking a harm reduction and trauma-informed approach to care. They also explain the importance of supplying cultural components in birthing by providing access to traditional cultural practices for pregnant and birthing women. They also describe the complexity of socioeconomic barriers facing the expectant Indigenous women and the ways in which they deal with them.

The Indigenous doulas place emphasis on connecting with mothers in the face of hostile medical and social systems. They recount their experiences as advocates and as a “buffer” to help empower women to make good decisions that are beneficial to them and their baby. The lack of confidence stems from ongoing trauma the pregnant Indigenous women experience. The doulas also feel that it is important to acknowledge that they need to be the right “fit” with their client. The doula collectives vary in their approaches to figuring out which doula would work best with which expectant Indigenous mother.

The interviews reveal that the work of Indigenous doulas is really about a cultural resurgence and a recognition that the current systems in place cause harm and damage to Indigenous women. The systemic racism that Indigenous expectant women face both in medical and social service settings continues to have deleterious outcomes. As one participant states, “every Indigenous family who wants a doula should have one.” Until we have medical systems and social services that no longer perpetuate racism, Indigenous doulas have an integral role to play.

## Data Availability

Data sharing is not applicable to this article as no datasets were generated or analysed during the current study.
